# Circulating tumour DNA and risk of recurrence in patients with asymptomatic versus symptomatic colorectal cancer

**DOI:** 10.1038/s41416-024-02867-5

**Published:** 2024-10-10

**Authors:** Nadia Øgaard, Sarah Østrup Jensen, Mai-Britt Worm Ørntoft, Christina Demuth, Mads Heilskov Rasmussen, Tenna Vesterman Henriksen, Jesper Nors, Amanda Frydendahl, Iben Lyskjær, Marijana Nesic, Christina Therkildsen, Jakob Kleif, Mikail Gögenur, Lars Nannestad Jørgensen, Jesper Vilandt, Jakob Benedict Seidelin, Kåre Anderson Gotschalck, Claudia Jaensch, Berit Andersen, Uffe Schou Løve, Ole Thorlacius-Ussing, Per Vadgaard Andersen, Thomas Kolbro, Alessio Monti, Jeppe Kildsig, Peter Bondeven, Nis Hallundbæk Schlesinger, Lene Hjerrild Iversen, Morten Rasmussen, Ismail Gögenur, Jesper Bertram Bramsen, Claus Lindbjerg Andersen

**Affiliations:** 1 Department of Molecular Medicine, Aarhus University Hospital, Aarhus, Denmark; 2 Institute of Clinical Medicine, Faculty of Health, Aarhus University, Aarhus, Denmark; 3 Department of Surgery, Gødstrup Hospital, Herning, Denmark; 4 Gastro Unit, Surgical Section, Copenhagen University Hospital, Amager-Hvidovre, Denmark; 5 Department of Clinical Medicine, University of Copenhagen, Copenhagen, Denmark; 6 Center for Surgical Science, Department of Surgery, Zealand University Hospital, Køge, Denmark; 7 Digestive Disease Centre, Bispebjerg Hospital, Copenhagen, Denmark; 8 Department of Surgery, Nordsjællands Hospital, Hillerød, Denmark; 9 Department of Gastroenterology and Hepatology, Herlev Hospital, Herlev, Denmark; 10 Department of Surgery, Horsens Hospital, Horsens, Denmark; 11 Department of Public Health Programs and University Research Clinic for Cancer Screening, Randers Regional Hospital, Randers, Denmark; 12 Department of Surgery, Viborg Hospital, Viborg, Denmark; 13 Clinical Cancer Research Center, Aalborg University, Aalborg, Denmark; 14 Department of Surgery, Odense University Hospital, Odense, Denmark; 15 Department of Surgery, Odense University Hospital, Svendborg, Denmark; 16 Department of Surgery, North Denmark Regional Hospital Hjørring, Hjørring, Denmark; 17 Department of Surgery, Copenhagen University Hospital, Herlev, Denmark; 18 Department of Surgery, Regional Hospital Randers, Randers, Denmark; 19 Department of Surgery, Aarhus University Hospital, Aarhus, Denmark

**Keywords:** Colorectal cancer, Prognostic markers, Tumour biomarkers

## Abstract

**Background:**

Multiple initiatives aim to develop circulating tumour DNA (ctDNA) tests for early cancer detection in asymptomatic individuals. The few studies describing ctDNA-testing in both asymptomatic and symptomatic patients report lower ctDNA detection in the asymptomatic patients. Here, we explore if asymptomatic patients differ from symptomatic patients e.g. by including a ‘low-ctDNA-shedding’ and ‘less-aggressive’ subgroup.

**Methods:**

ctDNA assessment was performed in two independent cohorts of consecutively recruited patients with asymptomatic colorectal cancer (CRC) (Cohort#1: *n* = 215, Cohort#2: *n* = 368) and symptomatic CRC (Cohort#1: *n* = 117, Cohort#2: *n* = 722).

**Results:**

After adjusting for tumour stage and size, the odds of ctDNA detection was significantly lower in asymptomatic patients compared to symptomatic patients (Cohort#1: OR: 0.4, 95%CI: 0.2–0.8, Cohort#2: OR: 0.7, 95%CI: 0.5–0.9). Further, the recurrence risk was lower in asymptomatic patients (Cohort#1: sHR: 0.6, 95%CI: 0.3–1.2, Cohort#2: sHR: 0.6, 95%CI: 0.4–1.0). Notably, ctDNA-negative asymptomatic patients had the lowest recurrence risk compared to the symptomatic patients (Cohort#1: sHR: 0.2, 95%CI: 0.1–0.6, Cohort#2: sHR: 0.3, 95%CI: 0.2–0.6).

**Conclusions:**

Our study suggests that asymptomatic patients are enriched for a ‘low-ctDNA-shedding-low-recurrence-risk’ subgroup. Such insights are needed to guide ctDNA-based early-detection initiatives and should prompt discussions about de-escalation of therapy and follow-up for ctDNA-negative asymptomatic CRC patients.

## Introduction

Cancer is a major health concern and is one of the leading causes of mortality worldwide [1]. For most patients the diagnosis of cancer is prompted by symptoms which often means that the diagnosed disease is at an advanced stage. To reduce cancer mortality, early detection is imperative as mainly patients with low-stage disease can be offered curative treatment [2].

In recent years, multiple academic groups and companies have strived to explore the use of tumour-agnostic circulating tumour DNA (ctDNA) testing for early cancer detection, ultimately in asymptomatic individuals [3–14]. However, most ctDNA tests have only been investigated in symptomatic patients, while the performance in asymptomatic patients remains largely unexplored. The few studies investigating ctDNA test performance in both symptomatic asymptomatic individuals nearly all report a lower sensitivity for detecting asymptomatic cancers [4–6, 8, 14]. These observations now raise the question if the asymptomatic cancer population differs from the symptomatic cancer population beyond generally having smaller and less advanced tumours, e.g. by including a yet unappreciated subgroup of patients whose tumours are ‘low-ctDNA-shedders’ and less aggressive. This study aims to explore this critical question.

To assess ctDNA in asymptomatic versus symptomatic patients it is necessary to investigate a cancer type where a fraction of the patients is identified through screening and hence is asymptomatic. Colorectal cancer (CRC) is such a cancer type as population-based screening for CRC has been implemented in multiple countries [15–17]. In this study, we use CRC as a model system.

Here, we employed both tumour-agnostic methylation-based and tumour-informed mutation-based approaches to explore ctDNA detection in asymptomatic and symptomatic patients. Further, we aimed to investigate if the asymptomatic patients included a subgroup whose tumours were less aggressive compared to tumours from symptomatic patients.

## Materials and methods

### Patients and study design

The study included treatment-naïve patients with asymptomatic and symptomatic CRC from two independent cohorts, Cohort#1 and Cohort#2. Cohort#1 also included non-cancer controls. Plasma from all study subjects was retrospectively analysed for ctDNA blinded to the individual’s outcome (Supplementary Fig. S1).

Plasma from Cohort#1 subjects (215 patients with asymptomatic CRC, 117 patients with symptomatic CRC, and 804 non-cancer controls) was analysed using a tumour-agnostic ctDNA detection method querying three CRC-specific methylation markers by droplet digital PCR (ddPCR). Cohort#1 patients and non-cancer controls were recruited from 2014–2020 at the Surgical Departments of Aarhus, Randers, Horsens, Herning, Viborg, Bispebjerg, Hvidovre, Hillerød, Herlev, and Slagelse Hospitals. The symptomatic patients were all diagnosed with CRC after referral for diagnostic work up due to symptoms. The asymptomatic patients and the non-cancer controls were participants in the Danish CRC-screening programme. The asymptomatic patients were all diagnosed by colonoscopy after a positive faecal immunochemical screening test (FIT). The FIT test was scored as ‘positive’ at >100 ng haemoglobin/mL buffer corresponding to ≥20 µg haemoglobin/g faeces. The non-cancer controls included both FIT-negative individuals (*n* = 305) and FIT-positive (*n* = 499) individuals where no cancer was detected upon colonoscopy. The Danish CRC screening programme encourages individuals to participate only if they are healthy and asymptomatic. If screening invitees experience CRC-related symptoms the programme guidelines urge them to contact a physician directly. Thus, most patients with CRC diagnosed through screening are asymptomatic and will throughout the study be referred to as ‘asymptomatic’. All healthcare services and screening participation is free of charge in Denmark. This limits the risk of inclusion bias from socio-economic status among both asymptomatic and symptomatic CRC patients.

To ensure that Cohort#1 results were not biased by a difference in methylation patterns between tumours from patients with asymptomatic and symptomatic CRC and to validate the Cohort#1 findings, an independent cohort of 1,090 asymptomatic (*n* = 368) and symptomatic (*n* = 722) patients with CRC (Cohort#2) was analysed using an orthogonal tumour-informed mutation-based method. Cohort#2 patients were consecutively recruited and had treatment-naïve pre-operative plasma collected during 2018–2021 as part of the clinical trial IMPROVE (ClinicalTrials.gov NCT03637686).

After surgery, patients were offered follow-up according to the Danish national guidelines [18] recommending computed tomography (CT) scans at 12 and 36 months after surgery. For some patients (*n* = 133) no CT scan results were available. This was due to: (1) the follow-up shorter than 12 months (*n* = 63), (2) the patient died before month 12 (*n* = 13), (3) the patient was not offered follow-up due to old age (>80 years) (*n* = 53), or (4) the patient declined any follow-up (*n* = 4). Since our main aim of the study was to investigate differences in ctDNA detection complete follow-up was not an inclusion criterion. Patients without follow-up were excluded from recurrence analysis (see “Statistical analysis” section).

Due to the study’s explorative design, no sample size estimation (power analysis) was performed.

### Blood collection and processing

The same standard operating procedure was used for collection and processing of all blood samples included in this study (Supplementary Appendix 1). In brief, blood was collected in BD Vacutainer K2 EDTA tubes (Becton Dickinson) and processed within 2 h from venipuncture. A two-step plasma isolation procedure using double centrifugation at 3000 g for 10 min at 21 °C was applied to avoid contamination from hematopoietic cells. Isolated plasma was stored in cryotubes (Techno Plastic Products AG) at −80 °C until the time of circulating cell-free DNA (cfDNA) extraction.

### cfDNA isolation and quantification

cfDNA was extracted from 8 mL plasma on the QIAsymphony robot using the QIAamp Circulating Nucleic Acids kit (Qiagen) or manually using the QIAamp Circulating Nucleic Acid Kit (Qiagen) following manufacturer’s instructions. cfDNA was quantified by ddPCR (Bio-Rad Laboratories, Hercules, CA, USA) as previously described [19–21]. cfDNA purification efficiency and contamination with DNA from lysed lymphocytes were estimated by ddPCR [19, 22]. For details, see Supplementary Appendix 1.

### Methylation-based ctDNA detection

In Cohort#1, cfDNA was sodium bisulfite converted prior to ctDNA analysis using the EZ-96 DNA Methylation-Direct™ MagPrep kit (Zymo Research) either manually or automated on a Zephyr robot (for details, see Supplementary Appendix 1). ctDNA was quantified by a methylation-specific multiplex ddPCR test, targeting the promoter regions of *C9orf50*, *CLIP4*, and *KCNQ5*, and a cytosine-free quantification assay (the CF assay), described in Supplementary Appendix 1. Details regarding marker selection, development of the methylation assays, assay optimisation, and test and validation of assay performance (sensitivity and specificity) in case-control plasma cohorts are thoroughly described in our previous work [19, 20].

### Mutation-based ctDNA detection

In Cohort#2, ctDNA was quantified using a tumour-informed mutation-based ddPCR strategy as described by Henriksen et al. [23, 24]. In brief, whole exome sequencing (WES) of paired tumour and normal samples was performed and mutational clonality and multiplicity were assessed as previously described [25]. For each patient, a single clonal somatic mutation was selected and a ddPCR assay targeting this was designed and validated before the assay was applied to patient plasma. For details regarding the selection of mutational targets, assay development, and test and validation of assay sensitivity and specificity, please refer to Supplementary Appendix 1 and our previous work [23–25].

### Droplet digital PCR analysis

All ddPCR experiments were conducted on the Droplet Digital PCR System (Bio-Rad) according to manufacturer’s instructions and are reported in agreement with the guideline for reporting on Quantitative Digital PCR Experiments (dMIQE) (Supplementary Table S1) [26]. Raw fluorescence intensity data for all individual droplets in each well was extracted using Quantasoft (v1.7.4; Bio-Rad).

For methylation assays, thresholds for positive and negative samples were set as previously described [19, 20]. A sample was classified as ‘ctDNA positive’ if at least two of three methylation markers showed a positive signal, otherwise, the sample was classified as ‘ctDNA negative’. The R code for defining the plate-wise thresholds for positive and negative droplets and for calculating the concentration of methylated DNA is available at GitHub [27].

For the mutation-based assays, the detection of ctDNA was done according to a previously established method using the ddPCR-calling tool CASTLE available at GitHub [23]. Patient plasma samples with a CASTLE p-value below 0.01 were called ‘ctDNA positive’. For details see Supplementary Appendix 1.

### Statistical analysis

Subgroups were compared using Fisher’s exact test for discrete data of small sample size and Pearson’s Chi-squared test for multiple subgroups. For continuous data, a comparison of unmatched groups was performed using Wilcoxon rank sum test. Univariable and Multivariable logistic regressions were used to estimate odds ratios (OR) and 95% intervals of confidence (95% CI) to evaluate the association of clinical variables on ctDNA detection (outcome variable). The univariable regression included variables expected to affect ctDNA shedding, according to Table 1: tumour size, depth of invasion (pT), lymph node status (pN), distant metastasis status (pM), age, and location (confounder variables). The Multivariable regression included the variables significant in the univariable regression.

**Table 1:** Characteristics Cohort#1.

Variable^a^	Asymptomatic CRC *n* = 215	Symptomatic CRC *n* = 117	*P*-value^b^
**ctDNA result**			**<0.001**
Negative	108 (50%)	21 (18%)	
Positive	107 (50%)	96 (82%)	
**Age (years)**^**c**^	68 (64–73)	72 (66–79)	**<0.001**
**UICC Stage**			**<0.001**
I	91 (42%)	28 (24%)	
II	63 (29%)	38 (32%)	
III	49 (23%)	49 (42%)	
IV	12 (5.6%)	2 (1.7%)	
**pT category (pN0, pM0)**			**<0.001**
pT1	48 (31%)	0 (0%)	
pT2	43 (28%)	28 (42%)	
pT3	60 (39%)	36 (55%)	
pT4	3 (1.9%)	2 (3.0%)	
**pT category (pN1-2, M0)**			**0.047**
pT1	6 (12%)	0 (0%)	
pT2	8 (16%)	5 (10%)	
pT3	26 (53%)	34 (70%)	
pT4	8 (16%)	10 (20%)	
Unknown	1 (2%)	0 (0%)	
**pM category**			0.221
pM0	76 (35%)	92 (78%)	
pM1	12 (6%)	2 (2%)	
Unknown	127 (59%)	23 (20%)	
**Tumour size (mm)**^**c**^	30 (20–45)	50 (32–60)	**<0.001**
**Tumour location**			**<0.001**
Right	57 (27%)	56 (48%)	
Left	158 (73%)	61 (52%)	
**Histological subtype**			0.052
Adenocarcinoma	198 (93%)	99 (85%)	
Mucinous adenocarcinoma	13 (6.1%)	16 (14%)	
Other	4 (1.4%)	2 (1.7%)	

^a^*N* (%).

^b^Statistical differences of variables in asymptomatic vs. symptomatic patients. Significance was assessed using Pearson’s Chi-squared test; Wilcoxon rank sum test; and Fisher’s exact test. *CRC* colorectal cancer, *ctDNA* circulating tumour DNA, *IQR* interquartile range. *P*-values <0.05 were considered statistically significant and are marked as bold.

^c^Median (IQR).

Recurrence detection was assessed from the day of surgery and until: radiological recurrence (event), death (competing event for cumulative incidence functions of recurrence and for competing risk regression of recurrence), or until end of follow-up (censoring). Patients with less than 12 months of follow-up and no CT scan results were excluded from recurrence analyses. Cumulative incidence functions (CIF) of recurrence were constructed using the Aalen-Johansen estimator. Subdistributional hazard ratios (sHR) with 95%CI for recurrence were estimated using Fine&Gray regression and adjusted for pTNM and tumour size. *P*-values < 0.05 were considered significant (two-sided). All data analysis and statistical analyses were performed using R software versions 4.0.3 and 4.1.1.

## Results

The study included two cohorts, Cohort#1 and Cohort#2, each consisting of consecutively recruited screening and non-screening detected CRC patients (Supplementary Fig. S1). Furthermore, Cohort#1 included non-cancer controls.

Initially, we analysed 1,336 plasma samples from Cohort#1 comprising 215 asymptomatic CRC patients (62% male), 117 symptomatic CRC patients (54% male), and 804 non-cancer controls (63% male, median age: 69, interquartile range (IQR): 63–74). Clinical characteristics for the cancer patients of Cohort#1 are summarised in Table 1. Asymptomatic patients were slightly younger (median age: 68, IQR: 64–73) than the symptomatic patients (median age: 72, IQR: 66–79). These were also diagnosed with lower Union for International Cancer Control (UICC) stages than the symptomatic patients including pT1 stage I and III tumors which were not observed for the symptomatic patients. Tumours from asymptomatic patients were smaller than tumours from symptomatic patients (median 30 mm vs. 50 mm, *P* < 0.001) and a larger fraction of tumours were left-sided in asymptomatic patients (44% vs. 23%, *P* < 0.001).

### ctDNA detection in patients with asymptomatic vs. symptomatic CRC (Cohort#1)

Plasma samples from all Cohort#1 study subjects were analysed for presence of ctDNA using a well-established tumour-agnostic methylation-based ddPCR test [19, 20, 28].

For the non-cancer controls 13 out of 804 (1.6%) samples were classified as ctDNA positive corresponding to a specificity of 98%.

**Fig. 1:**
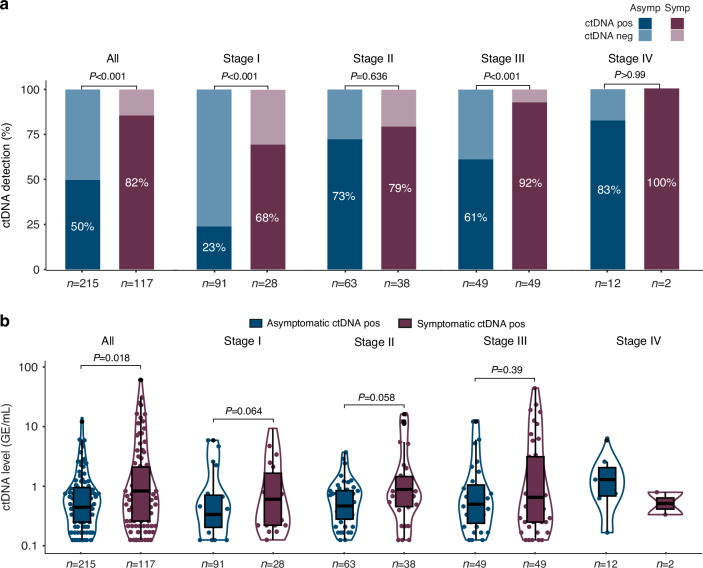
ctDNA detection in patients with asymptomatic and symptomatic CRC. ctDNA detection assessed by a methylation-based approach in plasma from asymptomatic and symptomatic patients in Cohort#1, **a** for all patients and stratified for UICC stages (I-IV). **b** ctDNA levels for ctDNA-positive samples for all patients and stratified for UICC stages. Statistical differences in ctDNA levels between asymptomatic and symptomatic patients were estimated by Wilcoxon rank sum (only if *n* > 4 in both subgroups) *P*-values < 0.05 are considered as statistically significant. *Asymp*: asymptomatic, *Symp*: symptomatic, *GE*: genome equivalents, *ctDNA*: circulating tumour DNA, *CRC*: colorectal cancer.

For CRC patients, treatment-naïve pre-operative plasma was used for ctDNA analysis. Overall, the ctDNA-positive fraction was substantially lower for asymptomatic patients than for symptomatic patients (50% vs. 82%, *P* < 0.001, Table 1 and Fig. 1a). When the patients were stratified by UICC stage (Fig. 1a) or pT/pN categories (Supplementary Fig. S2) the ctDNA-positive fraction remained lower for the asymptomatic patients. When assessing the ctDNA-positive samples the overall ctDNA levels (methylated genome equivalents (GE) per mL plasma) were significantly lower for patients with asymptomatic CRC compared to patients with symptomatic CRC (*P* = 0.018, Fig. 1b). The same trend was present when the patients were stratified by UICC stages. The lower ctDNA detection rate observed in the asymptomatic patients was not confounded by the overall plasma cfDNA levels as there was no difference between the cfDNA levels in asymptomatic vs. symptomatic patients (*P* = 0.51) (Supplementary Fig. S3).

### Regression analysis of ctDNA status in patients with asymptomatic vs. symptomatic CRC

Univariable and Multivariable logistic regressions were performed to evaluate the association of clinical variables on ctDNA detection. The univariable regression included variables expected to affect ctDNA shedding (“Statistical analysis” and Table 1): tumour size, depth of invasion (pT), lymph node status (pN), distant metastasis status (pM), age, and location (confounder variables). The variables: subgroup (symptom status), pT, pN, and tumour size were significant in this regression and were included in the multivariable regression. In the multivariable regression, after adjustment for pT, pN, and tumour size, the odds of detecting ctDNA remained lower for asymptomatic than for symptomatic patients (OR: 0.43, 95%CI: 0.22–0.81, *P* = 0.011, Table 2).

**Table 2:** Univariable and Multivariable logistic regression of ctDNA detection stratified by subgroup, adjusted for age, pTNM categories, tumour size, and location.

Characteristic	Cohort#1 *n* = 332						Cohort#2 *n* = 1090					
	Univariable			Multivariable			Univariable			Multivariable		
	OR^a^	95% CI^a^	*P*-value	OR^a^	95% CI^a^	*P*-value	OR^a^	95% CI^a^	*P*-value	OR^a^	95% CI^a^	*P*-value
**Exposure variable**												
**Subgroup**												
Symptomatic CRC	1	(ref)		1	(ref)		1	(ref)		1	(ref)	
Asymptomatic CRC	0.22	0.12–0.37	**<0.001**	0.43	0.22–0.81	**0.011**	0.42	0.33–0.54	**<0.001**	0.68	0.51–0.92	**0.013**
**Confounder variables**												
**Age**	1.01	0.98–1.03	0.624				1.01	1.00–1.03	0.140			
**pT category**												
pT1	1	(ref)		1	(ref)		1	(ref)		1	(ref)	
pT2	5.35	2.42–12.9	**<0.001**	1.01	0.38–2.77	0.988	8.20	3.92–20.1	**<0.001**	4.09	1.91–10.2	**<0.001**
pT3	19.7	9.14–46.6	**<0.001**	1.13	0.38–3.44	0.823	25.1	12.3–60.5	**<0.001**	5.80	2.68–14.5	**<0.001**
pT4	18.3	6.12–62.8	**<0.001**	0.56	0.12–2.71	0.472	39.0	16.2–107	**<0.001**	5.39	1.98–16.3	**0.002**
**pN category**												
pN0	1	(ref)		1	(ref)		1	(ref)		1	(ref)	
pN1–2	3.03	1.81–5.19	**<0.001**	1.50	0.77–2.97	0.241	2.35	1.72–3.26	**<0.001**	1.83	1.27–2.67	**0.001**
**pM category**												
pM0	1	(ref)										
pM1	3.99	1.06–25.9	0.073									
**Tumour size (mm)**	1.08	1.06–1.10	**<0.001**	1.07	1.05–1.10	**<0.001**	1.06	1.05–1.07	**<0.001**	1.05	1.04–1.06	**<0.001**
**Tumour location**												
Right	1	(ref)					1	(ref)				
Left	1.01	0.63–1.60	0.982				1.04	0.82–1.32	0.768			

^a^*OR* Odds Ratio, *Cl* Confidence Interval, *P*-values < 0.05 were considered statistically significant and are marked as bold.

To confirm the observations and to exclude that they were confounded by asymptomatic and symptomatic cancer having distinct methylation patterns, we validated the results in an independent cohort (Cohort#2) using a tumour-informed mutation-based ctDNA detection method. Cohort#2 comprised 368 asymptomatic and 722 symptomatic patients with UICC stage I-III CRC (Supplementary Fig. S1, and Supplementary Table S2). The multivariable regression analysis of Cohort#2 corroborated the findings from Cohort#1, i.e. after adjusting for the same confounding variables (pT, pN, and tumour size) the odds of detecting ctDNA in plasma from asymptomatic patients was significantly lower compared to symptomatic patients (OR: 0.68, 95% CI: 0.51–0.92, *P* = 0.013, Table 2).

### Risk of recurrence in patients with asymptomatic vs. symptomatic CRC

Given the lower ctDNA detection rate in the asymptomatic patients and that ctDNA detection generally is associated with poorer prognosis, we speculated if the asymptomatic patients had a lower risk of recurrence.

A comparison of the 3-year CIFs of recurrence for asymptomatic and symptomatic patients revealed a lower CIF recurrence rate for the asymptomatic patients (Cohort#1: 10.2% vs. 18.8%, Cohort#2: 14.0% vs. 24.2%) (Supplementary Fig. S4). Competing risk regression analysis, with death as competing event and adjusted for pT, pN, pM, and tumour size, confirmed a lower risk of recurrence in asymptomatic patients (Cohort#1: sHR: 0.55, 95%CI: 0.25–1.21 and Cohort#2: sHR: 0.60, 95% CI: 0.40–1.02) (Table 3).

**Table 3:** Competing risk regression of recurrence, stratified by subgroup, adjusted for pTNM categories and tumour size.

Characteristic	Cohort#1 *n* = 328^a^			Cohort#2 *n* = 939^a^		
	sHR^b^	95% CI^b^	P-value	sHR^b^	95% CI^b^	*P*-value
**Exposure variable**						
**Subgroup**						
Symptomatic CRC	1	(ref)		1	(ref)	
Asymptomatic CRC	0.55	0.25–1.21	0.076	0.60	0.40–1.02	**0.029**
**Confounder variables**						
**pT category**						
pT1	1	(ref)		1	(ref)	
pT2	2.72	0.31–24.2	0.4	1.03	0.35–3.02	>0.9
pT3	5.16	0.67–39.5	0.11	1.16	0.40–3.39	0.8
pT4	9.12	1.04–80.0	**0.046**	3.34	1.07–10.5	**0.039**
**pN category**						
pN0	1	(ref)		1	(ref)	
pN1–2	3.51	1.70–7.21	**<0.001**	4.25	2.75–6.56	**<0.001**
**pM category**						
pM0	1	(ref)				
pM1	4.41	1.65–11.8	**0.003**			
**Tumour size (mm)**	1.00	0.98–1.01	0.7	1.00	0.99–1.01	0.5

Subdistributional hazard ratios (sHR) of recurrence were estimated using Fine-Gray regression with death from any cause as competing event, *P*-values < 0.05 were considered statistically significant and are marked as bold.

^a^ Patient follow-up and eligibility for recurrence analysis is described in the “Patients and study design” and “Statistical analysis” sections.

^b^*sHR* subdistributional hazard ratio, *CI* confidence interval, *CRC* colorectal cancer.

### ctDNA status and risk of recurrence in patients asymptomatic vs. symptomatic CRC

**Fig. 2:**
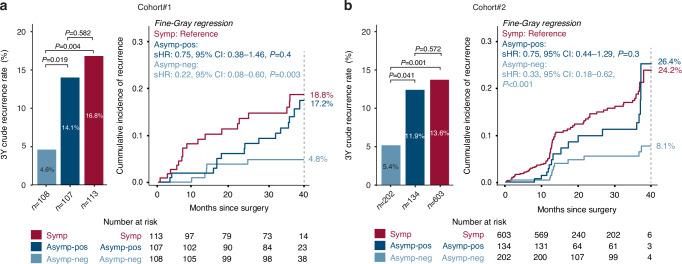
Recurrences and ctDNA status in patients with asymptomatic and symptomatic CRC. Crude 3-year recurrence rates and cumulative incidence functions (CIF) of recurrence, with death as competing event, stratified for ctDNA status for asymptomatic and symptomatic patients, for **a** Cohort#1, where follow-up data was available from 328 of the patients, hereof 39 with recurrence and 289 without recurrence (median follow-up: 36 months, IQR: 26–40). **b** Cohort#2, where follow-up data was available from 939 of the patients, hereof 109 with recurrence and 830 without recurrence. Patients were offered follow-up visits according to national guidelines with CT-scans at approximately 12 and 36 months after surgery. For some patients the 36-month scan was delayed for up to 40 months after surgery, and therefore all recurrences diagnosed up to 40 months after surgery are included in the 3-year recurrence rates. Patient follow-up and eligibility for recurrence analysis are described in the “Patients and study design” and “statistical analysis” sections. *P*-values for recurrence rates were calculated using Fisher’s exact test. CIFs were generated using the Aalen-Johansen estimator, CIF-estimated recurrence rates at 40months are given at the dashed line. sHR and 95%CI were estimated by Fine-Gray regression. *Asymp-neg*: asymptomatic ctDNA negative, *Asymp-pos*: asymptomatic ctDNA positive, *symp*: symptomatic, *sHR*: subdistribution hazard ratio, *CI*: confidence interval, *ctDNA*: circulating tumour DNA, *IQR*: interquartile range.

To investigate if ctDNA status could add further detail to the risk of recurrence for patients with asymptomatic CRC, we compared the 3-year crude recurrence rates of ctDNA-positive and ctDNA-negative asymptomatic patients to the 3-year crude recurrence rate of the symptomatic patients (Fig. 2a, b). The ctDNA-negative asymptomatic patients had significantly lower recurrence rates than both ctDNA-positive asymptomatic patients and the symptomatic patients (Cohort#1: 4.6% vs. 14.1% and 16.8%, respectively, Cohort#2: 5.4% vs. 11.9% and 13.6%, respectively). This was corroborated by the CIF-estimated recurrence rates (Cohort#1: 4.8% vs. 17.2% and 18.8%, respectively, Cohort#2: 8.1% vs. 24.2% and 26.4%, respectively). Assigning the recurrence risk of the symptomatic patients as ‘reference’ revealed that the risk of recurrence for ctDNA-negative asymptomatic patients was only 20–30% of that of symptomatic patients (Cohort#1: sHR: 0.22, 95%CI: 0.08–0.60, *P* = 0.003, Cohort#2: sHR: 0.33, 95%CI: 0.18–0.62, *P* < 0.001) (Fig. 2a, b). In contrast, the recurrence risk of ctDNA-positive asymptomatic patients was similar to the recurrence risk of the symptomatic patients (Cohort#1: sHR: 0.75, 95% CI: 0.38–1.46, *P* = 0.4, Cohort#2: HR: 0.75, 95% CI: 0.44–1.29, *P* = 0.3).

## Discussion

In recent years, enormous efforts have been invested to explore the use of tumour-agnostic ctDNA testing for early detection of cancer, ultimately in asymptomatic individuals. However, the performance of ctDNA-detection methods has mainly been evaluated in symptomatic patients. Consequently, the performance in asymptomatic patients remains largely unexplored. To address this we compared the ctDNA status of asymptomatic and symptomatic CRC patients in two different cohorts.

The observed ctDNA-positive fraction in the symptomatic patients (82%) aligned with our previously reported sensitivities for symptomatic patients with stage I-IV CRC (74–91%) [19, 20]. However, in the asymptomatic patients, the overall test sensitivity decreased to 50%. Similar observations have previously been reported for the FDA-approved CRC screening test Epi proColon. When the Epi proColon assay was applied to samples from a screening-like cohort a lower sensitivity (48.2%) was observed compared to the sensitivity in the initial cohort ofsymptomatic patients (67%) [4, 29].

The lower ctDNA levels attributed to asymptomatic patients have previously been linked to their lower UICC stage and smaller tumour size compared to symptomatic patients (stage migration) [13, 30]. Our study verified the association between lower stage, smaller tumour size, and reduced ctDNA detection rate. Notably, a novel insight from our data was that even after adjusting for pT, pN, and tumour size, ctDNA detection rates remained lower in the asymptomatic patients. Since Cohort#1 was analysed by a methylation-based ctDNA detection approach, we speculated if this observation could be attributed to differences in methylation patterns between tumours in asymptomatic vs. symptomatic patients. To address this, we validated our findings using an orthogonal tumour-informed mutation-based ctDNA detection method. Application of this method to a large independent cohort confirmed a significantly lower ctDNA detection rate in asymptomatic vs. symptomatic patients after adjusting for pT, pN, and tumour size.

The persistently lower ctDNA detection rate in plasma from asymptomatic patients indicates that their tumours may phenotypically differ from tumours from symptomatic patients beyond size, depth of invasion, and lymph node spread, in ways that impact the tumour’s ability to shed ctDNA to the circulation.

We hypothesised that the absence of symptoms combined with a low-shedding phenotype could be indicative of a less aggressive CRC subgroup. Using recurrence risk as a surrogate for aggressiveness this hypothesis was supported by our discovery that asymptomatic patients had a significantly lower recurrence risk compared to symptomatic patients, even after adjusting for tumour pTNM and size. The finding is further corroborated by a recent Danish population-based cohort study, which demonstrated a lower 5-year risk of recurrence in screening-detected (asymptomatic) compared to non-screening-detected CRC (symptomatic) [31].

Furthermore, our results showed that it is particularly the ctDNA-negative asymptomatic patients, not the ctDNA-positive, that define the CRC-subgroup with improved prognosis. This is consistent with recent evidence from Chen et al. [3]. who reported increased overall survival to be associated with lower ctDNA shedding across various cancer types. They also found that cancer patients identified through screening had better overall prognosis. Collectively, these results suggest that ctDNA-negative asymptomatic patients may belong to a less aggressive CRC subgroup than the classical symptomatic CRC patients.

These results raise the question if the predicted survival benefit of population-based CRC screening might be overestimated. If screening leads to diagnosis of a ‘novel’ non-aggressive CRC subtype which due to the asymptomatic phenotype had not previously been identified through the symptom-driven diagnostic work-up, then overestimation would be a likely consequence. Our data suggests that integrating ctDNA into the assessment of screening-detected cancer could help identify this low-risk patient subgroup (asymptomatic ctDNA-negative patients) and that it may be justified sparing this subgroup from adjuvant chemotherapy and standard-of-care follow-up. Further clinical studies are needed to explore these implications.

Given the indications of different ctDNA shedding phenotypes among CRC patients, studies addressing potential biological features as predictors of ctDNA shedding and tumour aggressiveness are highly warranted. We hypothesise that differences in vascularity, fibrosis, proliferative activity, and apoptosis rates may be root-causes for the low and high ctDNA-shedding phenotypes. Further studies are needed to unravel the role of these and other biological features in defining the ctDNA shedding phenotypes [32, 33]. Furthermore, studies investigating whether the presence of symptoms in general or specific symptoms correlates with ctDNA shedding will be crucial in understanding why symptomatic patients are more likely to shed ctDNA than asymptomatic patients.

Tumours from asymptomatic patients with a ‘low-ctDNA-shedding’ phenotype pose a potential challenge for implementing blood-based ctDNA screening for early cancer detection. To address this, several preventive measures can be considered. Sensitivity can be enhanced by increasing the number of ctDNA markers analysed, as demonstrated in recent studies using emerging ultra-sensitive ctDNA detection methods [8, 12, 34] or by applying a multi-omics strategy in combination with machine learning [7, 11]. However, even the most sensitive tumour-agnostic methods currently available will struggle to robustly detect ctDNA when cfDNA tumour fraction is below 0.01% [35]. Consequently, patients with asymptomatic CRC are likely to go undetected if the ctDNA level is below this limit. Moreover, if the low ctDNA shedding for asymptomatic patients is a generic characteristic across cancer types, this could explain why MCED tests, e.g. the Galleri test, exhibit poorer performance in detecting cancers identified through screening than detecting cancers identified due to symptoms [6, 8]. It has previously been proposed that ctDNA tests detect the clinically most significant cancers [32], which we confirm by our discovery that ctDNA-negative asymptomatic patients have a reduced risk of recurrence. Nonetheless, early-detection ctDNA tests should be optimised and validated in asymptomatic patients for optimal performance in this setting.

A few study limitations need to be acknowledged. When patients were stratified for pTNM, tumour size, and age, the subgroups became small increasing the 95% CIs and the risk of a type II error. This was exemplified when we investigated patient recurrence risk (Table 3). Here, results were only significant for Cohort#2 which was three times larger than Cohort#1. Nevertheless, the sHRs for the two independent cohorts were highly similar and therefore, our findings robustly support that asymptomatic patients have a better prognosis than symptomatic patients.

Further, it can be argued that if a more sensitive ctDNA test had been used the number of ctDNA-positive asymptomatic patients would have been higher. However, a more sensitive test would also increase the number of ctDNA-positive symptomatic patients and therefore the proportion of ctDNA-positive asymptomatic and symptomatic patients would expectedly remain unchanged.

In conclusion, we show that asymptomatic patients differ from symptomatic patients by having tumours characterised by a ‘low-ctDNA-shedding’ phenotype and by having lower risk of recurrence. Such insights are needed to guide the expectations of initiatives exploring ctDNA approaches for early cancer detection and should prompt discussions about de-escalation of adjuvant therapy and follow-up for ctDNA-negative asymptomatic CRC patients.

## Supplementary information

Suppl. Figures S1-S4

Suppl. Table S1

Suppl. Table S2

Supplementary Appendix 1

## Data Availability

The data generated in this study are not publicly available due to patient privacy requirements but are available upon reasonable request to the corresponding author.
