# Supplementary material for: Circulating tumour DNA and risk of recurrence in patients with asymptomatic versus symptomatic colorectal cancer
Source: Br J Cancer. 2024 Oct 10;131(10):1707–15. doi: 10.1038/s41416-024-02867-5 (PMC11555384; doi:10.1038/s41416-024-02867-5)
Supplement: Supplementary file 1 — Suppl. Figures S1-S4 [file 41416_2024_2867_MOESM1_ESM.pdf]

Suppl. Figure S1

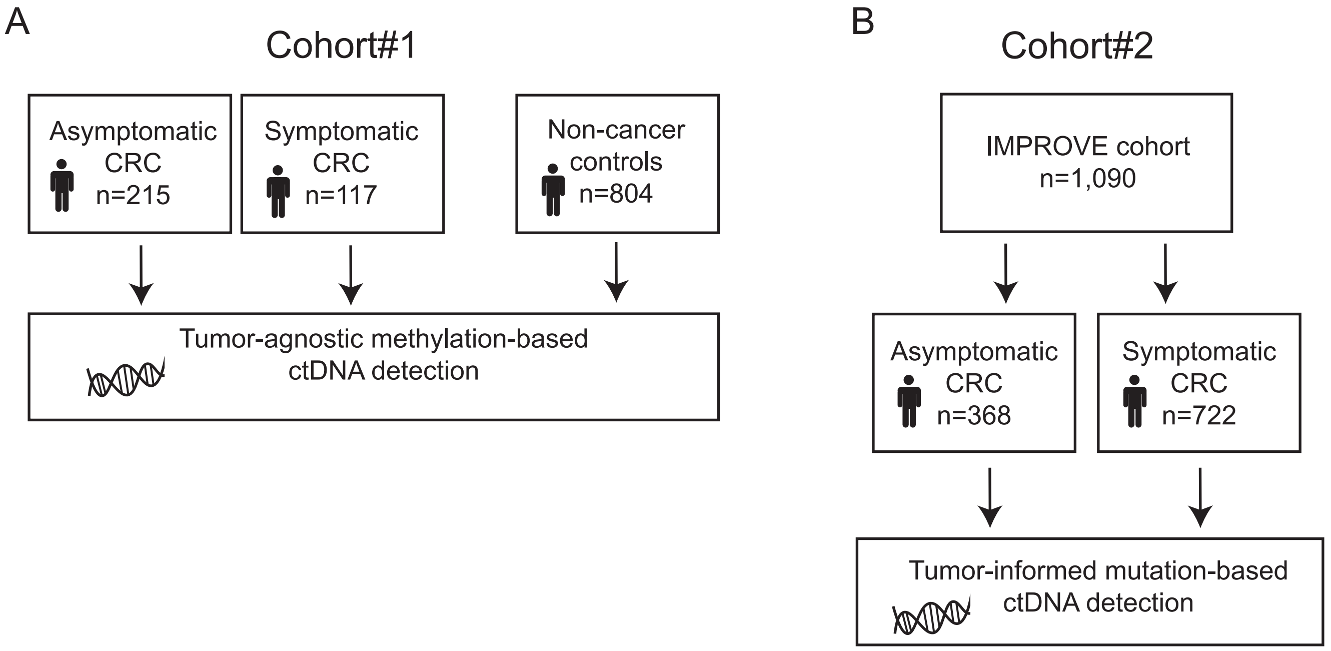

Suppl. Figure S1: Flowchart: A) Cohort#1 includes patients with asymptomatic CRC (n=215), symptomatic CRC (n=117), and non-cancer controls (n=804). The symptomatic patients were consecutively included and were diagnosed after referral for diagnostic workup due to symptoms. The asymptomatic patients and non-cancer controls were participants from the Danish CRC-screening program. The diagnosis of the asymptomatic patients was prompted by a positive FIT screening test and CRC detected upon subsequent colonoscopy. Samples in Cohort#1 were analyzed by methylation-based ctDNA detection. **B)** Cohort#2 includes patients who were recruited consecutively after diagnosis of CRC during 2018-2021 as part of the clinical trial IMPROVE. The cohort included both symptomatic patients where the CRC diagnosis was prompted by symptoms and asymptomatic patients diagnosed through CRC screening. Samples in Cohort#2 were analyzed by mutation-based ctDNA detection. ctDNA: circulating tumor DNA, CRC: colorectal cancer, FIT: fecal immunochemical test.

Suppl. Figure S2

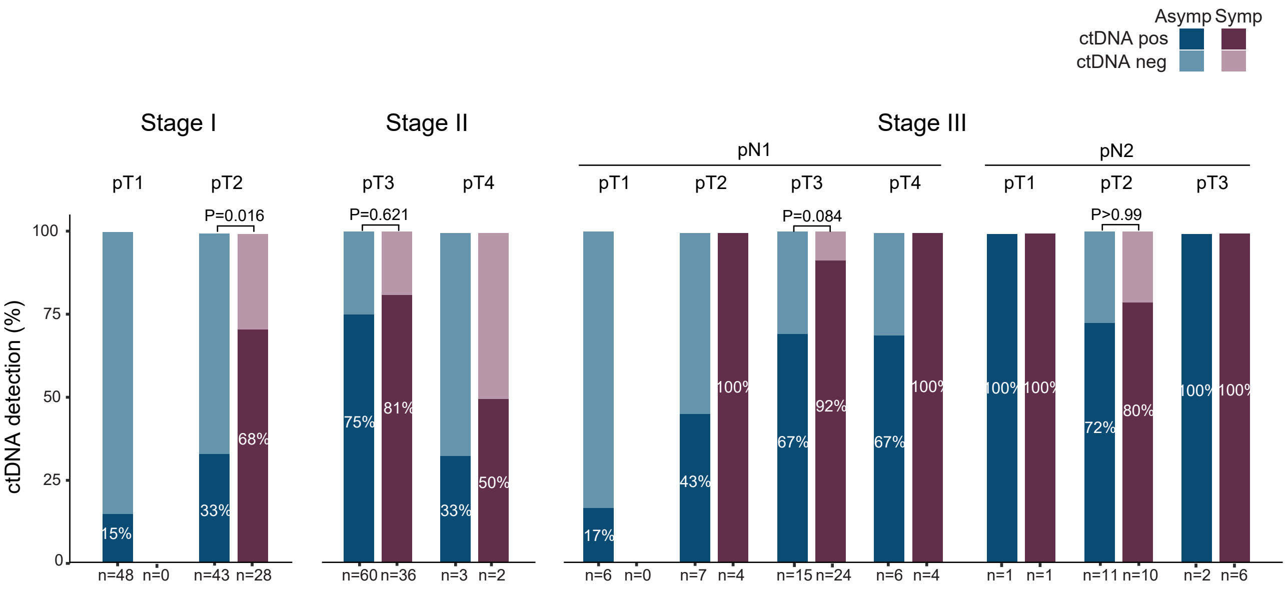

Suppl. Figure S2: ctDNA detection in patients with asymptomatic and symptomatic CRC stratified for pT and pN. ctDNA detection in Cohort#1 in all patients (215 asymptomatic CRC patients, and 117 symptomatic CRC patients), stratified for stages and for pN and pT catagories. Statistical differences in ctDNA detection between asymptomatic and symptomatic CRCs were estimated by Fisher's Exact test. Only subgroups with n>4 patients in each group were considered for statistical analysis. P-values <0.05 were considered statistically significant. Asymp: asymptomatic, Symp: symptomatic, ctDNA: circulating tumor DNA, pT: pathological tumor category, pN: pathological lymph node category, CRC: colorectal cancer.

Suppl. Figure S3

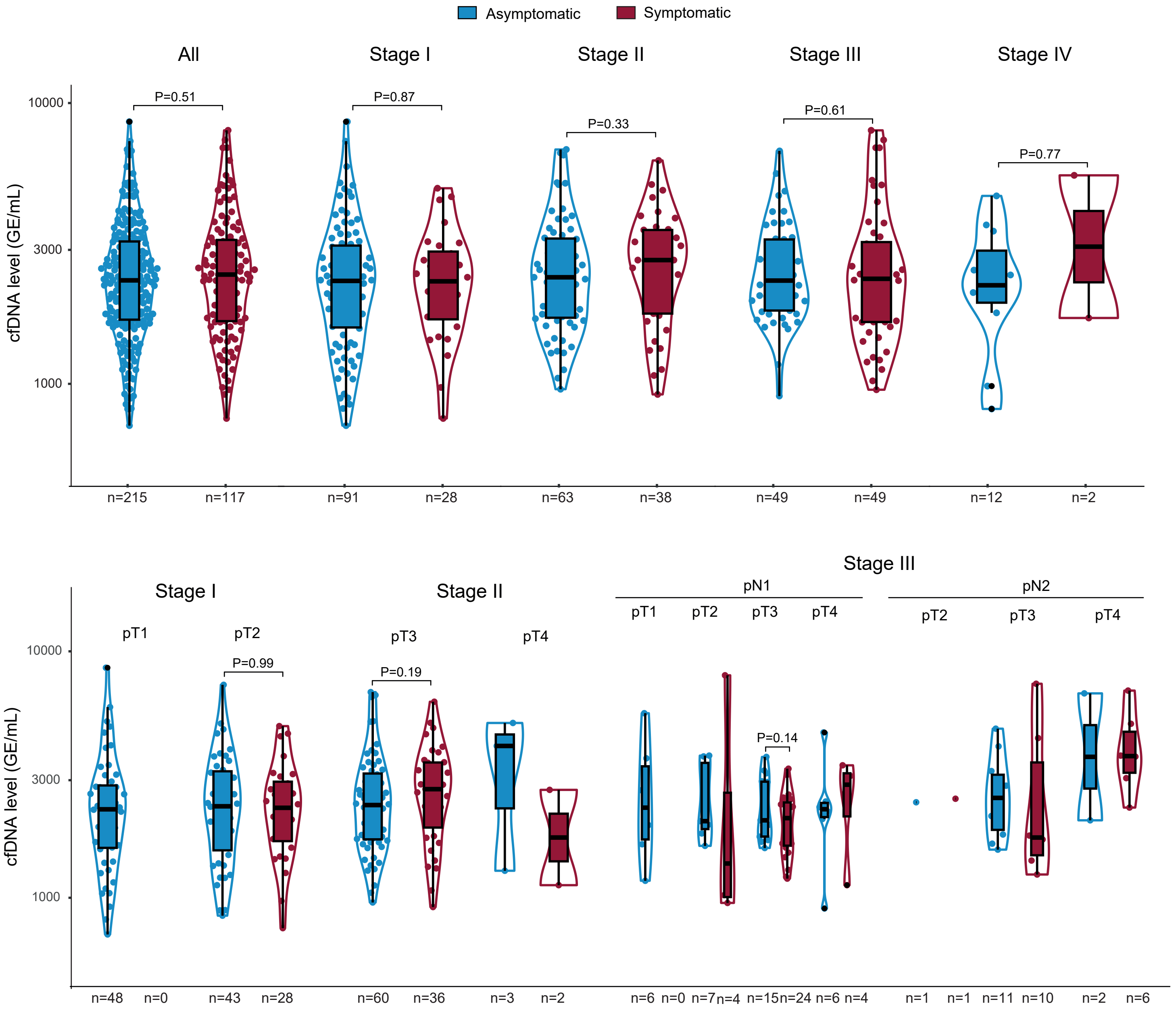

Suppl. Figure S3: circulating cell-free DNA levels. cfDNA levels (GE per mL plasma) in samples used for ctDNA analyses (Cohort#1) in all patients (215 asymptomatic CRC patients, and 117 symptomatic CRC patients), and stratified for stages. Differences in cfDNA levels between groups were estimated by Wilcoxon rank sum test (only if n>4 in both subgroups), P-values <0.05 are considered statistically significant. cfDNA: circulating cell-free DNA, ctDNA: circulating tumor DNA, CRC: colorectal cancer, GE: genome equivalents.

Suppl. Figure S4

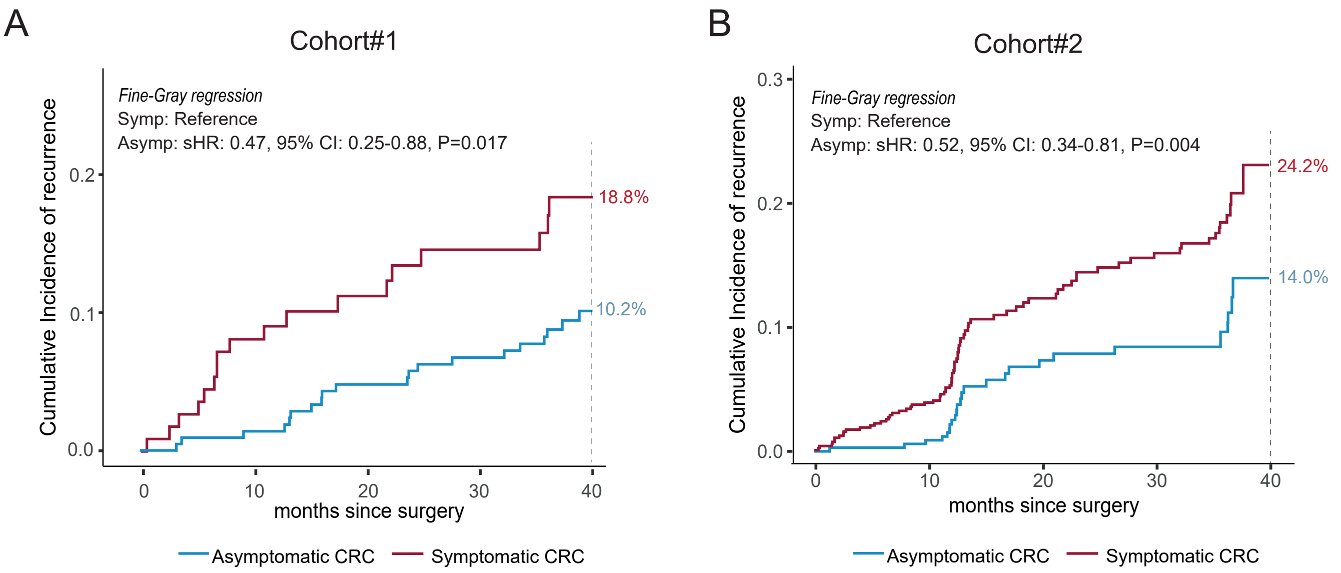

At Risk

Asymp	215	207	189	182	61
Symp	113	97	79	73	28

At Risk

Asymp	336	331	175	162	7
Symp	603	570	247	205	6

Suppl. Figure S4: Cumulative incidence of recurrence. Cumulative incidence functions (CIF) of recurrence (using the Aalen-Johansen estimator) for asymptomatic and symptomatic patients with death from any cause as a competing event for: **A)** Cohort#1, and **B)** Cohort#2. sHRs were estimated using Fine-Gray regression. Patients were offered follow-up visits according to national guidelines with CT-scans at approximately 12 and 36 months after surgery. Patient follow-up and eligibility for recurrence analysis is described in the “Patients and study design” and “Statistical analysis” sections. CRC: colorectal cancer, CI: confidence interval, sHR: subdistribution hazard ratio.
