# Supplementary material for: Circulating tumour DNA and risk of recurrence in patients with asymptomatic versus symptomatic colorectal cancer
Source: Br J Cancer. 2024 Oct 10;131(10):1707–15. doi: 10.1038/s41416-024-02867-5 (PMC11555384; doi:10.1038/s41416-024-02867-5)
Supplement: Supplementary file 3 — Suppl. Table S2 [file 41416_2024_2867_MOESM3_ESM.docx]

**Suppl. Table S2: Patient characteristics, Cohort#2**

| **Variable^1^** |  | **Asymptomatic CRC**,  n = 368 | **Symptomatic CRC**,  n = 722 | **P-value**^3^ | |
| --- | --- | --- | --- | --- | --- |
| **ctDNA result** |  |  |  | <0.001 | |
| negative |  | 221 (60%) | 280 (39%) |  | |
| positive |  | 147 (40%) | 442 (61%) |  | |
| **Age^2^** |  | 67 (61-72) | 75 (66-80) | <0.001 | |
| **UICC stage** |  |  |  | <0.001 | |
| I |  | 162 (44%) | 191 (26%) |  | |
| II |  | 141 (38%) | 381 (53%) |  | |
| III |  | 65 (18%) | 150 (21%) |  | |
| **pT (pN0)** |  |  |  | <0.001 | |
| pT1 |  | 54 (18%) | 42 (7.3%) |  | |
| pT2 |  | 108 (36%) | 149 (26%) |  | |
| pT3 |  | 138 (46%) | 354 (62%) |  | |
| pT4 |  | 3 (1.0%) | 27 (4.7%) |  | |
| **pT (pN1-2)** |  |  |  | 0.030 | |
| pT1 |  | 1 (1.5%) | 1 (0.7%) |  | |
| pT2 |  | 14 (22%) | 16 (11%) |  | |
| pT3 |  | 43 (66%) | 98 (65%) |  | |
| pT4 |  | 7 (11%) | 35 (23%) |  | |
| **Tumor size (mm)** |  | 30 (20-40) | 40 (27-56) | <0.001 | |
| **Tumor location** |  |  |  | <0.001 | |
| right |  | 139(38%) | 367 (51%) |  | |
| left |  | 229 (62%) | 355 (49%) |  | |
| ^1^n (%), ^2^Median (IQR), ^3^ Statistical differences of variables in asymptomatic vs. symptomatic patients were estimated using: Pearson's Chi-squared test; Wilcoxon rank sum test; and Fisher's exact test. | | | | |  |
|  | | | | |  |
