# Supplementary material for: Circulating tumour DNA and risk of recurrence in patients with asymptomatic versus symptomatic colorectal cancer
Source: Br J Cancer. 2024 Oct 10;131(10):1707–15. doi: 10.1038/s41416-024-02867-5 (PMC11555384; doi:10.1038/s41416-024-02867-5)
Supplement: Supplementary file 4 — Supplementary Appendix 1 [file 41416_2024_2867_MOESM4_ESM.docx]

**Supplementary Appendix 1**

**1. Supplementary Materials and Methods, Cohort#1**

**Sample collection**

Blood from all patients was collected before surgery. Blood collection and plasma isolation (within 2 hours) were performed according to the Standard operating procedure (SOP), attached below. Plasma was stored at -80⁰C until the time of cfDNA isolation.

**cfDNA isolation and quantification**

Plasma was thawed at room temperature and cfDNA was extracted from 8 ml plasma on the QIAsymphony robot using the QIAamp Circulating Nucleic Acids kit (Qiagen, protocol CF2000_CR2598_ID506_V1), or manually using the QIAamp Circulating Nucleic Acid Kit (Qiagen) following manufacturer’s instructions. Purified cfDNA was eluted in LoBind 96-well plates or single LoBind tubes (Eppendorf) in a volume of 60 µL and stored at -80°C until further use. cfDNA was quantified by ddPCR as previously described (1,2).

The median purification efficiency was 74% (interquartile range (IQR) 69-80%). The median number of accepted droplets (partitions) generated was 20,656 (IQR: 19,140-21,438). Only six samples showed contamination with lymphocyte DNA with >2 droplets being positive on ddPCR analysis. These samples were flagged rather than excluded.

**ddPCR assays**

cfDNA in plasma was quantified using an assay targeting a reference gene located on chromosome 1 (‘CF assay’), which rarely shows copy number alterations in cancer (3). This assay amplifies a cytosine-free genomic region which allows cfDNA quantification before and after sodium bisulfite conversion. Thus, the cfDNA recovery can be estimated as the ratio of ‘CF quantity’ before and after bisulfite conversion.

DNA purification efficiency and contamination with DNA from lysed lymphocytes were estimated as previously described (4).

Methylation-based ctDNA detection included specific primers and probes targeting fully methylated, bisulfite converted regions of the *C9orf50*, *KCNQ5*, and *CLIP4* genes. The identification, optimization and validation of the markers were described in previous studies (1,2). All samples were analyzed using two duplex reactions (*C9orf50* + *KCNQ5* and *CLIP4* + ‘CF-assay’), i.e. the sample volume was divided into two reactions.

**ddPCR setup**

All ddPCR experiments were conducted on the Droplet Digital PCR System (Bio-Rad) according to manufacturer’s instructions and the Minimum Information for Publication of Quantitative Digital PCR Experiments (dMIQE) guidelines (5). The reaction mix included 8 µL template cfDNA, 18 pmol forward primer, 18 pmol reverse primer, 5 pmol probe, 2x Supermix for Probes (no UTP; Bio-Rad) and AccuGENE™ Molecular Biology Water (Lonza) to a final volume of 22 µL. Droplets of 1 nL were generated on the QX200 AutoDG Droplet Generator (Bio-Rad). After droplet generation, the samples were amplified by PCR on a S1000 Thermal cycler (Bio-Rad) using the following program: 95⁰C for 10 minutes, 45 cycles of: 95⁰C for 30 seconds, and 56⁰C for 1 minute, and one final cycle of 98⁰C for 10 minutes. PCR products were stored at 4⁰C for up to 18 hours before analysis on a QX200 reader (Bio-Rad). Positive, negative, and no-template controls (NTC) were included for each assay. For methylation-based ctDNA detection, the positive control was 5 ng methylated bisulfite converted DNA, the negative control was 66 ng non-methylated bisulfite converted DNA (Zymo Research), and a water sample was NTC. No technical replication was performed.

**Sodium Bisulfite Conversion of cfDNA**

Before cytosine conversion, cfDNA was dried using vacuum centrifugation at 30°C (speedVac, Concentrator plus 5350, Eppendorf AG) and resuspended in 20 µL AccuGENE™ Molecular Biology Water (Lonza). cfDNA was sodium bisulfite converted using the EZ-96 DNA Methylation-Direct™ MagPrep kit (Zymo Research) either manually or automated on a Zephyr robot according to the manufacturer’s instruction, but with the following volumes of reagents: 60 µL CT conversion reagent, 280 µL M-Binding buffer, 5 µL MagBinding Beads, 185 µL M-Wash buffer, 93 µL M-Desulphonation Buffer, and 25 M-Elution Buffer. Fully methylated and fully unmethylated DNA control samples were included in all batches as positive and negative controls, respectively. Reactions were performed on an S1000 Thermal Cycler (Bio-Rad). All cfDNA was analyzed directly after bisulfite conversion. The median recovery after bisulfite-conversion was 28% (IQR: 20-35%).

**Methylation-based ctDNA analysis**

The raw fluorescence intensity data for all individual droplets in each well was extracted using Quantasoft v1.7 software (Bio-Rad) and analyzed plate-wise as previously described (1,2). Briefly, fluorescence data from a fully methylated positive control sample on each plate was used to identify fluorescence maxima for the negative and positive droplet populations. A gaussian kernel density was applied that identified exactly two maxima and one minimum. All samples on each plate were subsequently normalized to the median fluorescence of the negative population from the positive control. The fluorescence threshold for calling droplets positive or negative was finally set for all wells using a stringency parameter, beta, of 0.15, thus including 85% of data after the threshold point set at the minimum point between the negative and positive populations as defined by the positive control sample. The concentration c (copies per well) of methylated DNA was calculated as c = -*N**ln(1-P/*N*), where *N* is the total number of droplets and P is the number of positive droplets (6). The code in the R language is available at GitHub (7). A sample was classified as ‘ctDNA positive’ if at least two of three methylation markers showed a positive signal above the threshold, otherwise the sample was ‘ctDNA negative’. This algorithm was developed and validated in previous studies (1,2).

A median input of 3,960 genome equivalents (GE) (IQR: 2,236-5,100) of bisulfite converted cfDNA was used in each reaction. The median number of accepted droplets (partitions) generated was 16,006 (IQR: 14,982-17,056).

**2. Supplementary Materials and Methods, Cohort#2**

**Sample collection and DNA extraction**

Blood samples were collected and processed prior to surgery, for all patients with CRC included in IMPROVE, as described in the SOP for blood collection below.

Tumor biopsies were collected from the resected primary tumor (fresh frozen (FF) or formalin fixed and paraffin embedded tissue (FFPE)). Tumor DNA was extracted from FF tumor tissue samples using the Puregene DNA purification kit (Gentra Systems) and from FFPE samples with the QiAamp DNA FFPE tissue kit (Qiagen). Tumor and buffy coat DNA was quantified by the Qubit™ dsDNA BR Assay Kit (ThermoFisher).

Cell-free DNA was purified from 8 mL of plasma in single replicate using the QIAamp Circulating Nucleic Acids kit (Qiagen) or the QIAsymphony DSP Circulating DNA Kit (Qiagen) on the QIAsymphony robot (Qiagen). DNA was eluted in 60μL Suspension Buffer (Sigma). The cfDNA was quantified by ddPCR (Bio-Rad Laboratories), with assays targeting regions on chr3 and chr7 with little copy-number variation in CRC, as described previously (8). No extraction blanks were used.

**Whole exome sequencing**

Whole exome sequencing of paired tumor and buffy coat DNA samples were conducted as described in detail previously (9). Sequencing libraries were constructed with the Twist Library Preparation kit using enzymatic fragmentation (TWIST Bioscience). By standard protocol, libraries were prepared with xGen UDI-UMI Adapters. Libraries with DNA from buffy coat, FF tissue, and a subset of FFPE tissue samples were prepared with 50 ng input and 10 min fragmentation. Libraries with DNA from remaining FFPE tissue samples were prepared with 200 ng input and 6 min fragmentation. Library amplification was done with 7 or 8 cycles PCR. Using the NGS Human Core Exome (TWIST Bioscience, ~33 Mb), the exome was captured with hybridization probes. Target-enriched libraries were sequenced using the NovaSeq platform with 2x150 bp paired-end sequencing to a mean sequencing depth of 130x for FF tissue DNA, 150x for FFPE tissue DNA, and 60x for buffy coat DNA.

Raw sequencing reads were converted into FastQ files using Illumina bcl2fastq. Sequencing adapters were removed bioinformatically by cutadapt (v3.0) (10), and trimmed reads were mapped to the human reference genome (hg38) using BWA-MEM (v0.7.17) (11) with PCR duplicates flagged by Picard (v2.23.3) MarkDuplicates (12). Alignment was processed further using GATK (v4.1.9.0) BaseRecalibrator according to the GATK Best Practices (13). Germline variants were identified with GATK HaplotypeCaller (14), scored with GATK CNNScoreVariants (15), and filtered by GATK FilterVariantTranches (16). Somatic single nucleotide variants and small insertions/deletions were identified using GATK Mutect (17) and Strelka (18). Single-nucleotide polymorphism concordance between tumor and matched buffy coat DNA samples was examined to guard against sample swaps.

**dPCR assay design and optimization**

The dPCR assays were either custom designed in-house (ordered from Sigma Aldrich) or made-to-order from ThermoFisher. For custom assays, mispriming, GC-content, and optimal melting temperatures were checked by Primer3 (19). Sequences were checked for cross reactivity using In Silico PCR (20). All PCR amplicons were <150 bp in length, with most amplicons being <80 bp in length.

Assays were checked for linearity and sensitivity using a 4-point dilution series of tumor DNA in a fixed concentration of 3,030 genome equivalents (GE) of wild-type DNA per µL (mutant allele frequencies of: 1%, 0.3%; 0.1%; 0.03%). The optimal PCR cycling conditions were assessed by running the tumor dilution series on a five-point temperature gradient, with the optimal temperature selected based on linearity, droplet amplitude, and sensitivity.

**ddPCR setup**

All ddPCR reactions were prepared in a 22 µL reaction volume, with 20 µL converted to droplets (approx. 0.834 nL each) on the Automated Droplet Generator (Bio-Rad). Plasma samples were analyzed with up to 9µL template. High-concentration samples were run in a lower input volume (5µL) in multiple wells to avoid oversaturation. For plasma samples, a total of 54µL DNA eluate was divided into multiple ddPCR reactions, depending on the template volume. As all plasma samples were split across multiple wells, a high number of droplets could be generated for each sample (mean: 124,000 droplets, sd: 46,000 droplets). This resulted in a mean of 0.20 GE per droplet (sd: 0.22 GE/droplet). A water sample was included as a NTC. An assay-specific tumor DNA sample (3000-6000 GE) carrying the targeted mutation was included as positive control, and a pool of buffy coat DNA (6000 GE) from healthy donors was included as a negative control. Additionally, a patient-specific tumor (3000-6000 GE) and buffy coat (3000-6000 GE) DNA sample was run with a given patient’s plasma as positive and negative control, respectively. Mastermix was prepared with ddPCR Supermix for probes (No dUTP) (Bio-Rad, Cat:1863024) and a primer/probe mix (20x) in a 1:10 ratio. PCR reactions were run on the S1000 Thermal Cycler (Bio-Rad) and plates were kept at 12^o^C for a minimum of 5 hours to ensure maximum droplet stability. Droplets were analyzed on a QX200™ Droplet Reader (Bio-Rad). No technical replication was performed.

**ddPCR data processing**

Signal from the assay-specific positive control was used to set the threshold for positive droplets in the QuantaSoft software (v1.7.4, Bio-Rad). The threshold was fixed at a pre-determined number of standard deviations from the mean amplitude of the positive droplets. No signal should be observed in the NTC, and positive signal should be observed in the assay-specific and patient-specific positive controls. Reactions with droplet amplitudes outside the expected interval and droplet counts below 8000 were discarded. As plasma samples were split in multiple reactions, reaction-level results passing QC were merged to obtain the sample-level results.

**Mutation calling algorithms**

The CASTLE algorithm (21) was used to evaluate whether the mutation signal observed in the plasma sample was greater than what would be expected by noise. A thorough evaluation and explanation of all four algorithms have been published previously (21).

In brief, the expected noise was estimated by measuring the mutational signal in a five-point dilution series of fragmented non-mutated buffy coat DNA from 19 healthy donors. Three parameters representing different error sources were modeled based on the noise observed in healthy samples: the ɑ-parameter (arbitrary errors, the error rate of ɑ-errors is independent of the DNA concentration); the β-parameter (errors from a wild-type fragment giving off a mutational signal, i.e. PCR-induced errors. The error rate of β-errors is dependent on the DNA concentration in each droplet); the γ-parameter (errors of nontargeted probe-DNA interactions. The error rate of γ-errors is dependent on the DNA concentration in each droplet). Assuming the observed number of mutational DNA fragments in a droplet follow a Poisson distribution dependent on the DNA input, the true mutational signal was modeled using maximum likelihood theory, as the DNA concentration which – in addition to the expected errors – would result in the observed mutational signal. A likelihood ratio test was used to test if the estimated true mutational signal was greater than zero. A p-value<0.01 was considered significant.

**3. Standard Operating Procedure for blood collection (SOP)**

**ALL** blood samples must be collected in BD-tubes (Becton Dickinson), endotoxin-, RNAse- and DNAse-free.

**The following is necessary:**

1 blood collection system with safety features

1 tourniquet

1 tube with coagulation enhances for serum, 10 mL capacity, red cap (BD Vacutainer, ref. 367896)

7 EDTA-tubes, 10 mL capacity, purple cap (BD Vacutainer K2EDTA, ref. 367525)

1 centrifugation tubes, capacity 50 mL (sterile, DNAse- and RNAse-free) (CM-lab cat# 91050)

4 cryo tubes, capacity 10 mL (sterile, DNAse- and RNAse-free) (VWR cat# 479-1237)

8 cryo tubes, capacity 2 mL (sterile, DNAse- and RNAse-free) (TPP product# 89020)

4-5 manual sterile pipettes, capacity 5 mL

1 electronic pipette

2 pipettes for the automatically pipetting devices, capacity 5 and 25 mL (AL229206B, AL229226B)

**Collection of blood sample**

1. Prepare 7 EDTA-tubes and 1 tube with coagulation enhances for serum by applying labels with the patients CPR number and CRC biobank patient ID.
2. Apply stasis on the patient’s upper arm using the tourniquet, identify the best venipuncture site and puncture the vein with the needle.
3. Collect samples in the indicated order
   - 1 × 10 ml tube with coagulation enhances for serum (red cap)
   - 7 × 10 ml EDTA-tubes (purple cap)
4. Gently invert the tubes 8-10 times immediately after collection or place the tubes on a flipping device

Inverting or flipping decreases the risk of fibrin clotting in the collection tubes for serum and to mix EDTA and blood in the EDTA-tubes.

**Handling of samples**

**NB.**

After blood sampling, blood collection tubes must be centrifuged within 1,5 hours. Completion of all steps in the instruction is required within 2 hours.

Serum tubes needs incubation at room temperature until coagulated, this takes approximately 30 min. Hereby, the risk of haemolysis and formation of fibrin clots is minimised. Besides, the risk of haemolysis can be reduced by applying only light stasis during sampling and by sampling from a large vein. If the tube is not filled, risk of haemolysis can be reduced by adjusting the pressure in the tube. This is done by removing and then reapplying the rubber cap.

1. Centrifugation of plasma and serum tubes is performed at room temperature (21°C), 3.000*g* for 10 min. The centrifuge is set to slow deceleration, approximately 45 sec. This counteracts mixing of the separated phases. After initiation of the centrifugation, the process may not be stopped.
2. Prepare the following cryo tubes:
   - 4 x 10 mL cryo tubes for plasma **01-04**
   - 3 x 2 mL cryo tubes for plasma **05-07**
   - 3 x 2 mL cryo tubes for buffy coats **08-10**
   - 2 x 2 mL cryo tubes for serum **11-12**

**Plasma-EDTA, purple cap**

1. All plasma supernatants (from 7 x 10 mL EDTA-tubes) are transferred to the 50 mL centrifugation tube
   Plasma is harvested very close to the buffy coat, to ensure maximum plasma output. The procedure for harvesting buffy coats from EDTA-tubes is described in step 6.
2. Centrifugation of plasma in the 50 mL tubes is repeated (3.000*g* for 10 min at 21 °C). There will be approximately 30-35 mL plasma in the tube.
3. Plasma is transferred from the 50 mL tubes to 4 x 10 mL cryo tubes **01-04** and 3 x 2 mL cryo tubes **05-07**. To each 10 mL cryo tube, 8 mL plasma is added, and to each 2 mL cryo tube 2 mL plasma is added.

**Elaboration on transferring plasma to 10 mL cryo tubes**

1.
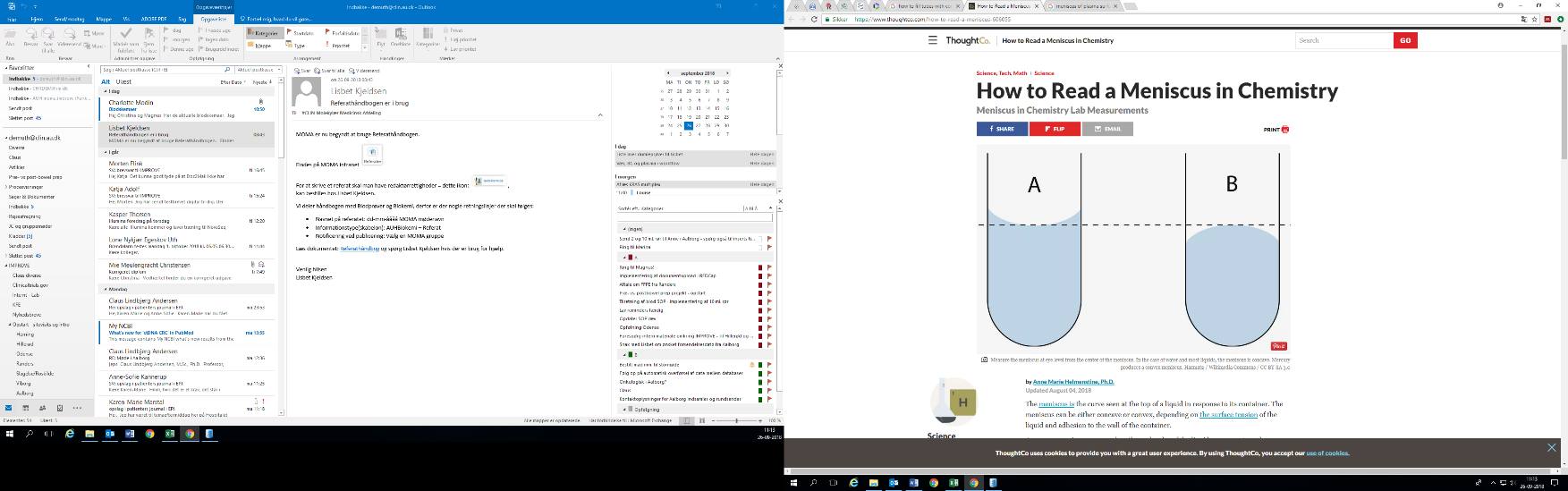
Plasma is added until the lowest plateau of plasma is in line with the 8 mL mark on the tube (se drawing). First, 8 mL is added to each of the 10 mL tubes (**01-04**). If the plasma volume is not sufficient for filling of 4 x 10 mL tubes, 8 mL is added to as many tubes as possible and the remaining plasma is added to the last 10 mL tube (also if less than 8 mL is remaining). If plasma remains after filling the 10 mL tubes, this is added to the 2 mL tubes (**05-07**). 2 mL plasma is added to as many tubes as possible (max. 3).
2. IMPORTANT: Do not touch the pellet in the 50 mL tube. This can contaminate the plasma and may affect the following analyses. Keep the pipette 0.5 cm above the pellet.

**Buffy coat, purple cap**

1. From 3 of the purple cap EDTA-tubes from step 3, buffy coats (thin white layer of cells on top of the red blood cells) are transferred to 3 x 2 mL cryo tubes **08-10**

**Serum, red cap**

1. Serum, from the red cap tube, is distributed equally in 2 x 2 mL cryo tubes **11-12**. To avoid contamination of the serum, do not touch the bottom 0.5 cm serum above the clot. If transferring serum from the tube is hindered by the presence of fibrin, the tube can be re-centrifuged at higher g for 10 min.

**Freezing**

1. All cryo tubes are frozen at -80 °C until further use.

**References**

(1) Jensen, S. O. et al. Novel DNA methylation biomarkers show high sensitivity and specificity for blood-based detection of colorectal cancer-a clinical biomarker discovery and validation study. Clinical epigenetics 11, 158, doi:10.1186/s13148-019-0757-3 (2019).

(2) Jensen, S. O., Ogaard, N., Nielsen, H. J., Bramsen, J. B. & Andersen, C. L. Enhanced Performance of DNA Methylation Markers by Simultaneous Measurement of Sense and Antisense DNA Strands after Cytosine Conversion. Clinical chemistry 66, 925-933, doi:10.1093/clinchem/hvaa100 (2020).

(3) Andersen, C. L. et al. Frequent genomic loss at chr16p13.2 is associated with poor prognosis in colorectal cancer. Int J Cancer 129, 1848-1858, doi:10.1002/ijc.25841 (2011)

(4) Pallisgaard, N., Spindler, K. L., Andersen, R. F., Brandslund, I. & Jakobsen, A. Controls to validate plasma samples for cell free DNA quantification. Clinica chimica acta; international journal of clinical chemistry 446, 141-146, doi:10.1016/j.cca.2015.04.015 (2015)

(5) M. G. & Huggett, J. F. The Digital MIQE Guidelines Update: Minimum Information for Publication of Quantitative Digital PCR Experiments for 2020. Clinical chemistry 66, 1012-1029, doi:10.1093/clinchem/hvaa125 (2020).

(6) Dube, S., J. Qin, and R. Ramakrishnan, Mathematical analysis of copy number variation in a DNA sample using digital PCR on a nanofluidic device. PLoS One, 2008. 3(8): p. e2876.

(7) <https://github.com/MOMA-CRC/ddanalyzor.git>

(8) Reinert T, Schøler LV, Thomsen R, Tobiasen H, Vang S, Nordentoft I, et al. Analysis of circulating tumour DNA to monitor disease burden following colorectal cancer surgery. *Gut*. 2016;65(4):625–634.

(9) Kabel J, Henriksen TV, Demuth C, Frydendahl A, Rasmussen MH, Nors J, et al. Impact of Whole Genome Doubling on Detection of Circulating Tumor DNA in Colorectal Cancer. *Cancers* . February 10, 2023;15(4). Available at: http://dx.doi.org/10.3390/cancers15041136

(10) Martin M. Cutadapt removes adapter sequences from high-throughput sequencing reads. *EMBnet.journal*. May 2, 2011;17(1):10–12.

(11) Li H. Aligning sequence reads, clone sequences and assembly contigs with BWA-MEM. 2013; arXiv:1303.3997v1 [q-bio.GN].

(12) Picard [Internet]. [cited June 25, 2021]. Available at: https://broadinstitute.github.io/picard/

(13) McKenna A, Hanna M, Banks E, Sivachenko A, Cibulskis K, Kernytsky A, et al. The Genome Analysis Toolkit: a MapReduce framework for analyzing next-generation DNA sequencing data. *Genome Res*. September 1, 2010;20(9):1297–1303.

(14) HaplotypeCaller [Internet]. [cited June 25, 2021]. Available at: https://gatk.broadinstitute.org/hc/en-us/articles/360037225632-HaplotypeCaller

(15) CNNScoreVariants [Internet]. [cited June 25, 2021]. Available at: https://gatk.broadinstitute.org/hc/en-us/articles/360047217991-CNNScoreVariants

(16) FilterVariantTranches [Internet]. [cited June 25, 2021]. Available at: https://gatk.broadinstitute.org/hc/en-us/articles/360051308071-FilterVariantTranches

(17) Mutect2 [Internet]. [cited June 25, 2021]. Available at: https://gatk.broadinstitute.org/hc/en-us/articles/360051306691-Mutect2

(18) Kim S, Scheffler K, Halpern AL, Bekritsky MA, Noh E, Källberg M, et al. Strelka2: fast and accurate calling of germline and somatic variants. *Nat Methods*. August 2018;15(8):591–594.

(19) Primer3Plus [Internet]. Available at: http://www.bioinformatics.nl/cgi-bin/primer3plus/primer3plus.cgi

(20) Kent J. UCSC In-Silico PCR [Internet]. Available at: https://genome.ucsc.edu/cgi-bin/hgPcr

(21) Henriksen TV, Drue SO, Frydendahl A, Demuth C, Rasmussen MH, Reinert T, et al. Error Characterization and Statistical Modeling Improves Circulating Tumor DNA Detection by Droplet Digital PCR. *Clin Chem*. January 14, 2022; Available at: http://dx.doi.org/10.1093/clinchem/hvab274
